# Emotional Intelligence and Perceived Health Related to Expressed Compassion Fatigue: A Study in Health Sector at Regional Level

**DOI:** 10.3389/fpsyg.2021.729624

**Published:** 2021-11-30

**Authors:** María Dolores Ruiz Fernández, María-Jesús Lirola, Juan Diego Ramos-Pichardo, Rocío Ortíz-Amo, Olivia Ibáñez-Masero, Susana Rodríguez Gómez, Ángela María Ortega-Galán

**Affiliations:** ^1^Department of Nursing, Physiotherapy, and Medicine, University of Almería, Almería, Spain; ^2^Facultad Ciencias de la Salud, Universidad Autónoma de Chile, Santiago, Chile; ^3^Department of Psychology, University of Almería, Almería, Spain; ^4^Department of Nursing, University of Huelva, Huelva, Spain; ^5^Department of Nursing Science, Physiotherapy and Medicine, University of Almería, Almería, Spain; ^6^Andusian Health Service, Seville, Spain

**Keywords:** burnout—professional, perceived health, quality of professional life, emotional well-being, fatigue, public health

## Abstract

**Background:** The work of health professionals often involves physical as well as psychological strain. They constantly deal with traumatic situations of pain and suffering, which destabilize the sense of well-being. Compassion fatigue is a feeling that appears in these cases and is related to other variables such as burnout or emotional drain.

**Aims:** The principal aim of this project was to deepen the analysis of compassion fatigue and how it could be explained through the relationship with other constructs such as emotional intelligence and perceived health.

**Methods:** This work followed the STROBE checklist for cross-sectional studies. In this study 1,521 nurses (*M_*age*_* = 47.32; *SD* = 8.44) participated. The responses reported by the nurses were analyzed by classifying them as high or low compassion fatigue and the differences of both groups were analyzed for the variables of emotional intelligence, perceived health and quality of professional life.

**Results:** It was obtained significant differences for all factors except for emotional intelligence factor. A linear regression analysis showed both emotional intelligence and perceived health helped to explain (12%) compassion fatigue.

**Conclusion:** This study provides light on comprehending the conception of compassion fatigue. It highlights the importance of intervention programs that improve the quality of professional life.

## Introduction

Healthcare requires professionals to be physically, emotionally and spiritually highly dedicated (Han et al., 2018), which is heightened by caring for people who are suffering or experiencing pain (Feeley et al., 2019). The repeated contact over time by professionals in traumatic contexts increases the risk of suffering physical and emotional problems (Hemsworth et al., 2020). The professional begins to experience anxiety, stress, and progressive wear and tear on a physical and emotional level (Kim, 2020). The consequences may be negative on their work and personal life, with the appearance of health problems (Parola et al., 2017).

The symptoms eventually developed by the healthcare provider are closely related to Compassion Fatigue (CF) syndrome (Roney and Acri, 2018). Compassion Fatigue is considered to be a phenomenon that occurs when professionals develop a diminished capacity for empathy, or compassion, as they deal daily with the discomfort or even the death of their patients (Peters, 2018). This concept is related to the secondary post-traumatic stress suffered by professionals, who in order to offer their help, experience the suffering and pain of their patients (Figley, 1995).

According to the current literature, a high percentage of health professionals end up suffering from Compassion Fatigue (Ortega-Campos et al., 2020). CF is considered one of the negative aspects of helping those who experience pain and suffering (Hemsworth et al., 2020). In contrast, as a positive aspect of helping patients is the compassion satisfaction, this is defined by a feeling of gratitude in caring for and relieving the pain of someone who is suffering (Jang et al., 2016). These constructs are measured through the Professional Quality of Life (QOL), understood as the sense of well-being that comes from the balance perceived by the individual and associated with the burden of the profession, in addition to the psychological, organizational, and relational resources that health professionals have to manage their emotional states derived from their working day (Rodarte-Cuevas et al., 2016). When measuring QoL, burnout is analyzed along with compassion fatigue and compassion satisfaction. Burnout is described as a pathological state of the “burnt-out” professional, arising from physical and psychological exhaustion due to experiences during and because of work. It has been shown that health professionals are at risk of suffering from compassion fatigue and burnout due to the characteristics of their work (Rivera-Ávila et al., 2017). In addition, the danger for the professional of suffering from any of these syndromes means that their general health is affected, taking into account that there is no evidence of a general (non-psychotic) psychiatric morbidity (Sabery et al., 2019). Most studies have emphasized the relevance of organizational factors and the appearance of syndromes such as CF (Sinclair et al., 2017) as predictive variables of the general health status observed in professionals (Turgoose and Maddox, 2017).

Notwithstanding, in the last decade, researchers have directed their focus toward those aspects of intelligence more linked to emotions and feelings (Crowne et al., 2017). They propose the idea that a person’s emotional intelligence (EI) may be a better predictor of work and social efficiency (Carragher and Gormley, 2017). Emotional intelligence as a multidimensional construct is defined as the ability to understand and manage their own emotions and other people’s emotions, as well as the ability to regulate and modify them (Kaya et al., 2018).

Health professionals are continually faced with psychological challenges in caring for people in critical situations (Borges et al., 2019). In particular, nursing professionals are the most exposed to direct contact with patients (Durkin et al., 2019). This is because nurses establish a relationship with patients and their families while trying to respond to a health demand (Shahar et al., 2019). When the desire to resolve this response is psychologically overwhelmed in facing suffering (de Oliveira et al., 2019) and the patient’s expectation, the nurse may experience anxiety, stress, and a progressive burnout (Gentry and Shockney, 2018).

Based on recent research it is known that emotional intelligence can be considered a moderating factor in nursing practice (White and Grason, 2019), and would influence the probability of developing symptoms such as stress (Huang et al., 2019) or suffering psychosocial risks leading to syndromes such as Compassion Fatigue (Amir et al., 2019). Nevertheless, studies relating emotional intelligence to compassion fatigue and the perceived health of nursing professionals are scarce. Therefore, the aim of this research was to examine the relation between emotional intelligence, perceived health and compassion fatigue.

## Materials and Methods

### Design

In this project, questionnaires were implemented in different health centers at a provincial level (Andalusia), following a cross-sectional design. The Strengthening the Reporting of Observational studies in Epidemiology was followed for the organization and structure of this investigation.

### Participants

The participants for this research corresponded to 1,521 nurses. The sample is representative of the Andalusian territory, all of them work in the public sector of the Andalusian community. The subjects according to official and public data from the Andalusian government on the total number of nurses working in 2015 (*N* = 22,533) show that the participants in this study offer an adequate number to be considered representative at the level of a 95% confidence interval and a 3% accuracy. The criteria established for participation in this study was the condition that the job performed involved direct contact with the patient. Thus, professionals working in areas such as administration, laboratories or management were excluded from the research.

### Instruments

A record of the **participants’ socio-demographic** variables was made, including information on gender and age.

#### Professional Quality of Life

Short form of ProQOL v.IV questionnaire has a 30-item self-report instrument designed to measure positive and negative effect of helping others, who experience suffering and trauma. Developed by Stamm (2005), and adapted to Spanish by Morante Benadero et al. (2006) and has been used in healthcare professionals (Buceta et al., 2019). This instrument has 30 items, 10 items to measure each of the three subscales that measure the positive and negative effects of helping people in suffering: (1) Compassion satisfaction (α = 0, 87)—satisfaction in doing the job by providing good to others and society; (2) Burnout (α = 0.72)—feeling of discouragement, hopelessness and that efforts at work make no difference; and (3) Compassion fatigue (α = 0.80)—state of stress from the indirect experience of another person’s trauma, constantly resuming images of trauma and trying to avoid situations that might remind one of the event. According to Galiana et al. (2017) scores higher or equal to 17 denote high compassion fatigue, medium 9–17, and low scores less than 8. Responses are recorded on a six-point Likert scale (0 = never; 1 = rarely; 2 = a few times; 3 = somewhat often; 4- often; 5 = very often).

#### Emotional Intelligence

##### Trait Meta-Mood Scale-24

The TMMS-24 (Fernandez-Berrocal et al., 2004) is a self-report test, its first English version was developed by Salovey et al. (2002). It is composed of 24 items from a 5-point Likert scale divided into three dimensions of EI: emotional perception or attention to feelings, emotional understanding or clarity, and emotional regulation or repair. Emotional attention refers to the awareness we have of our emotions, the ability to recognize our feelings and know what they mean. Emotional clarity: This refers to the ability to know and understand emotions, knowing how to distinguish between them, understanding how they evolve and integrating them into our thinking. Emotional repair: Refers to the ability to regulate and control positive and negative emotions. While high scores on clarity and repair are adequate, the same is not true of emotional attention, which can lead to hypervigilance of emotions and sensations, and consequently, hypochondriac (excessive fear and worry that some illness will be generated) can occur. Contrary to the assumption of many users of this questionnaire, the TMMS was not designed to provide an overall score, which should be taken into account when analyzing data and interpreting results derived from its application. This test consists of an internal consistency reported by its authors of α = 0.90 for perception, α = 0.90 in understanding and α = 0.86 for regulation of emotions.

#### General Health

##### General Health Questionnaire—12

The GHQ-12 is a 12-item, 4-point Likert-type scale that is frequently used as screening for psychological disorders (Goldberg and Williams, 1988; Spanish Version by Rocha et al., 2011). Respondents are asked to indicate the degree to which they have recently experienced a range of common symptoms of distress. The usual form of the GHQ score when used for case identification is the 0-0-1-1 method by Lobo and Muñoz (1996), where 0 is given for *Not at all* and *Same as usual*; and 1 is given for *More than usual*, and *Much more than usual*. Validation studies in 15 countries have found reliability results between alphas of 0.82 and 0.85 (Goldberg et al., 1997).

### Procedure

This research was organized at the regional level thanks to the participation of researchers from the eight provinces that make up Andalusia, Spain. The data collection was carried out in different health centers of the Public Health Service: a total of 48 information collection points among which are hospitals, and primary care centers. For this purpose, the principal investigators of this study contacted the management teams of the different healthcare centers in the Community of Andalusia to request their approval and collaboration in the study. Once the participating centers had been confirmed, the contacts of the heads or ward managers were provided to facilitate their instruction in the application of the questionnaires. Secondly, the participants were informed about the purpose of the study and in turn about the anonymous and voluntary nature of the study. Those who decided to participate gave their informed consent in writing. The Declaration of Helsinki marked throughout the course of the research the established ethical principles that were assumed and practiced. This research project followed the current national regulations on the protection of personal data (Organic Law 3/2018 on the Protection of Personal Data and the Guarantee of Digital Rights). The estimated time for completing the tests was 15 min.

### Data Analysis

Missing data were treated by a multiple imputation method. The first analysis conducted were the descriptive statistics. Secondly, the sample was analyzed for normality using the Kolmogorov-Smirnov test. According to the central limit theorem (Martínez González et al., 2020), parametric tests were carried out, albeit a number of variables did not comply with this criterion. Both parametric and non-parametric tests were convergent. Thirdly, different analyses were carried out to find out the relatedness among the diverse variables under study. In order to compare means, the Student’s *t*-test was used for independent samples. To determine the statistical significance, a threshold of 95% was established. The Pearson correlation was employed for the analysis of bivariate correlations. Finally, a linear stepwise regression model was created on the basis of the least-squares method. The dependent variable was the specific dimension of PRoQoL, compassion fatigue, and the explanatory variables were the different sub-dimensions of TMMS-24 (i.e., attention, clarity, and repair) and the measure of general health. A three-step linear regression analysis was performed to analyze the predictive power of the dimensions of emotional intelligence and general health on compassion fatigue. The Durbin-Watson statistic (1.64) was calculated, with values between 1.5 and 2.5 indicating independence of the residuals; and the values of tolerance and IVF as indicators of collinearity, with values close to 1 indicating no collinearity. Regression was controlled for gender and age The goodness of fit was obtained by using the determination coefficient (*R*^2^) and its fit version. Data treatment was performed with the statistical program SPSS V25 (IBM).

## Results

### Descriptive Statistics

Participants in this study ranged in age from 23 to 64 years old (*M*_*age*_ = 47.32; *SD* = 8.44). From the total of 1,521 participants, 75.5% were women, of whom 69.8% were married, and 59.5% had a stable employment situation. Most of the professionals worked in hospital environments (55%) and most of the centers were placed in city areas (89.5%). In terms of areas of work, 36.3% worked in the inpatient department, 23.6% in the critical and emergency department, 15.7% in the surgical area, 13.1% in pediatrics, and less than 12% for the support service, outpatient, mental health, palliative care, rehabilitation and others. Their most common working turns were the morning shift (32.1%) and the morning/afternoon/night shift (29%). Their average employment history was 275.36 months (*SD* = 110.42) and their average length of service was 146.81 months (*SD* = 117.52). The different descriptive statistics for each of the variables used in this study are shown in Table 1.

**TABLE 1:** Descriptive statistics for the total number of participants.

**Variable (instrument)**	**Subscales**	***N***	***M***	***SD***	**Skewness**	**Kurtosis**
TMMS-24	Attention	1,521	25.02	5.45	0.076	–0.113
	Clarity	1,521	27.63	5.39	0.156	–0.207
	Repair	1,521	29.43	5.33	–0.266	0.002
ProQol-IV	Satisfaction	1,521	35.48	7.40	–0.038	–0.899
	Burnout	1,521	23.44	5.29	–0.318	0.009
	Fatigue	1,521	20.74	7.88	–0.026	–0.636
GHQ	General health	1,521	0.16	0.19	1.734	3.349

*N, number of subjects; M, medium; SD, standard deviation; TMMS-24, Emotional Intelligence; ProQol-IV, Quality of professional life; GHQ, General Health.*

### Bivariate Analyses Between the Dimensions’ Object of Study

Correlation analysis (Table 2) showed positive associations between the three dimensions of the emotional intelligence scale, negative correlations for satisfaction in relation to burnout and fatigue, while satisfaction maintained a positive relationship with the variables of clarity and repair. Overall health showed a positive relationship with attention, burnout and fatigue and a negative correlation with clarity and repair.

**TABLE 2:** Bivariate correlations between the different variables under study.

**Variables**	**1**	**2**	**3**	**4**	**5**	**6**	**7**
1. Attention	−	0.29***	0.18***	0.03	0.07**	0.19***	0.10***
2. Clarity		−	0.54***	0.23***	−0.24***	−0.17***	−0.15***
3. Repair			−	0.13***	−0.17***	–0.30	−0.19***
4. Satisfaction				−	−0.42***	−0.49***	–0.03
5. Burnout					−	0.58***	0.31***
6. Fatigue						−	0.25***
7. General health							−

***p < 0.001; **p < 0.01.

### Differences Between the Two Populations With High and Low Fatigue

In Table 3 it can be seen the different means obtained for the different variables in a sample of people who present high compassion fatigue, observed means that show that attention, clarity and repair should be improved according to the instructions of the TMMS-24, the means for the dimensions of professional quality of life also show a low level and the average of general health (0.18) also reveals a low result. Also, it can be seen the different means obtained for a sample of people who present low compassion fatigue, observed means that show adequate attention, clarity and repair according to the instructions of the TMMS-24, the means for the dimensions of quality of professional life also show a high satisfaction and low burnout and fatigue; and the average of general health (0.05) the lack of detection of psychological disorders.

**TABLE 3:** Student’s *t*-test.

		***M* (*SD*)**	***t***	***p*-value**
Attention	High fatigue	25.48 (5.09)	5.47	0.000
	Low fatigue	22.24 (5.72)		
Clarity	High fatigue	26.94 (4.91)	–5.56	0.000
	Low fatigue	30.39 (6.01)		
Repair	High fatigue	29.22 (4.90)	–1.96	0.053
	Low fatigue	30.50 (6.36)		
Satisfaction	High fatigue	33.07 (6.69)	–12.93	0.000
	Low fatigue	42.11 (6.55)		
Burnout	High fatigue	25.15 (4.47)	17.43	0.000
	Low fatigue	16.88 (5.12)		
Fatigue	High fatigue	25.03 (5.27)	80.22	0.000
	Low fatigue	6.26 (1.67)		
General Health	High fatigue	0.18 (0.19)	10.26	0.000
	Low fatigue	0.05 (0.01)		

*M, mean; SD, standard deviation; t, Student’s t-test. Significance level p < 0.05.*

In addition, the differences between the means were significant with the exception of the repair variable whose *p*-value was 0.053. The group presenting high fatigue shows higher levels of attention, burnout, fatigue, and health, while the group manifesting low fatigue presents higher levels in clarity, repair, and satisfaction.

### Linear Regression Analysis

Table 4 reflects the results of predicting the variables of emotional intelligence and general health, controlling for gender and age. If we look at step two, the EI variables such as attention, clarity and repair helped explain 10% of compassion fatigue. Incorporation into the health model (step three) increased the explained variance of compassion fatigue by 12%. In fact, considering all the psychological variables considered in the model, it can be seen that clarity dimension was shown to be the greatest predictor of fatigue (β = −0.28, *p* < 0.001) and attention dimension (β = 0.22, *p* < 0.001).

**TABLE 4:** Linear regression analysis predicting compassionate fatigue from gender, age, emotional intelligence, and general health.

	***F***	***B* (β)**	***SE B***	***R* ^2^**	***t***	**Tolerance**	**FIV**
Step 1	3.72*			0.00	24.72		
Age		0.00 (0.06*)	0.00			0.996	1.004
Gender		−0.05 (−0.04)	0.04			0.996	1.004
Step 2	32.85***			0.10	15.94		
Age		0.01 (0.08**)	0.00			0.982	1.018
Gender		−0.04 (−0.03)	0.04			0.993	1.007
Attention		0.03 (0.25***)	0.00			0.895	1.117
Clarity		−0.03 (−0.30***)	0.00			0.895	1.117
Repair		0.01 (0.07*)	0.00			0.913	1.093
Step 3	34.41***			0.12	14.75		
Age		0.01 (0.08**)	0.00			0.982	1.018
Gender		−0.03 (−0.02)	0.04			0.988	1.012
Attention		0.03 (0.22***)	0.00			0.873	1.122
Clarity		−0.03 (−0.28***)	0.00			0.883	1.119
Repair		0.01 (0.10**)	0.00			0.899	1.115
General health		0.51 (0.16***)	0.08			0.930	1.075

F, Fisher-Snedecor test; B, non-standardized regression coefficients; β, standardized regression coefficients; SE B, standard error of B; R^2^, variance explained; t, Student’s t-test; *p < 0.05; ** p < 0.01; ***p < 0.001.

## Discussion

The aim of this research was to analyze the influence of emotional intelligence and perceived health on compassion fatigue. It has been observed that the three components of emotional intelligence (i.e., attention, clarity, and repair), along with perceived health influence compassion fatigue. Nurses with high levels of compassion fatigue use emotional care as a mechanism of emotional management and have poorer perceived health. However, participants with low levels of compassion fatigue experience emotional clarity and repair, resulting in better perceived health.

In this research the levels of compassion fatigue in nursing professionals are high, much higher than the average score obtained by Stamm (2005) in different groups. This result is consistent with other studies that have determined that nurses have high levels of compassion fatigue. The prevalence is even higher in services such as critical care, emergency medicine and oncology (Sacco et al., 2015; Ortega-Campos et al., 2020). Thus, among professionals working in services exposed to traumatic situations, pain and suffering (Durkin et al., 2019). On the basis of the fact that 55% of the nursing professionals in this study work in the hospital environment, this data could be aligned with these studies.

Emotional intelligence is a factor that may influence compassion fatigue, as found in this study, being a clear predictor of compassion fatigue, with the dimensions of clarity and attention being the best predictors. According to some studies, emotional intelligence is related to the management of job stress in nursing professionals (Nagel et al., 2016). Thus, it has been observed to improve emotional drain and burnout in health professionals (Al Barmawi et al., 2019). Furthermore, it is considered a moderating factor that could improve job satisfaction among nurses (Weng et al., 2011). Studies examining the role of emotional intelligence on compassion fatigue are scarce in health professionals. However, it has been observed that emotional intelligence is protective in the development of compassion fatigue (Amir et al., 2019). Compassion is the ability of human beings to respond to suffering, which means to understand the suffering of others and oneself and to respond with the actions necessary to alleviate it, which impacts on emotional satisfaction and well-being (Durkin et al., 2019). In order to provide adequate care, it is helpful to be compassionate with oneself, and professionals need to feel emotionally secure with themselves (Heffernan et al., 2010). With repeated exposure to painful or traumatic experiences, health professionals become emotionally numb, flooded and self-protective, and enter into a dysfunctional emotional loop. This is why the high compassion care nurses in this research are focused on emotional care and are not able to understand, or regulate, emotional states as they are in those nurses who have low compassion care fatigue. In Amir et al. (2019) research, mental health professionals who understood their emotions and were able to manage them decreased the likelihood of experiencing compassion fatigue.

In addition, compassion fatigue leads to nurses having a poorer perception of their overall health and health-related quality of life (Ruiz-Fernández et al., 2020). When the desire to alleviate suffering exceeds the possibility of being able to carry it out, it implies a psychological overstrain that culminates in anxiety, stress, and emotional burnout (Gentry and Shockney, 2018; de Oliveira et al., 2019). This is why nursing professionals with high levels of compassion fatigue in our research report a perceived poorer health.

The limitations of this study must be addressed. On the one hand, although it is a representative sample of the professionals who currently work in the Andalusian Public Health System (Spain), it is a mostly homogeneous sample because the participants were mostly middle-aged nurses, with a greater proportion of women and with extensive work experience. Furthermore, it is a transversal study, which allows the study of the relationships between the variables but not the establishment of cause and effect relationships between them. On the other hand, it is necessary to mention the risk of bias that is present due to the convenience sampling when filling out the questionnaires. It would be interesting for a better understanding of this topic to extend the study to a larger sample and to include new constructs related to compassion fatigue, such as clinical relevance and former experiences and empathy ability (Gleichgerrcht and Decety, 2013; Hunsaker et al., 2015).

## Conclusion

This study has a number of implications for health professionals and health services. Firstly, it has been observed that levels of compassion fatigue in nurses are very high. Second, that emotional intelligence and perceived health is highly related to compassion fatigue. Nurses with high levels of compassion fatigue have poorer perceived health and pay more attention to their emotions. The opposite is the case for professionals who have low compassion fatigue, who have better perceived health and who are able to understand and regulate emotional states. Therefore, health systems should offer intervention programs focused on cultivating or training compassion that include emotional regulation. Professionals who care for patients are exposed to stressful and traumatic situations. It cannot be overlooked that contact with suffering has certain psychological and physical implications, culminating in burnout and the emergence of certain syndromes in health professionals such as compassion fatigue. Research determines that cultivating compassion decreases the emotional cost of health professionals (Delaney, 2018). As an essential element, health professionals must be able to identify, understand and manage their own emotions and treat themselves with kindness through the exercise of self-compassion. In this way, emotional intelligence can help decrease the level of compassion fatigue and therefore protect mental health and emotional well-being. Therefore, it could be useful to introduce into nurses’ training programs the necessary content on coping, managing emotions or cultivating compassion and self-compassion that would allow nurses, from the beginning of their professional careers, to be able to effectively deal with the continuous contact with suffering.

## Data Availability Statement

The raw data supporting the conclusions of this article will be made available by the authors, without undue reservation.

## Ethics Statement

The studies involving human participants were reviewed and approved by the Research Ethics Committee of Almería Centro (CEI-27 September 2017), which was extended to the rest of Andalusia as a single authorization. The patients/participants provided their written informed consent to participate in this study.

## Author Contributions

MR and ÁO-G designed the research, to which the rest of the authors contributed (M-JL, JR-P, RO-A, OI-M, and SR). M-JL contributed to the data analysis. MR, ÁO-G, and M-JL wrote the first version of the manuscript, which was critically reviewed by all signatories, who approved the final version. All authors contributed to the interpretation of the results and critical review of the manuscript, and have read and agreed to the published version of the manuscript.

## Conflict of Interest

The authors declare that the research was conducted in the absence of any commercial or financial relationships that could be construed as a potential conflict of interest.

## Publisher’s Note

All claims expressed in this article are solely those of the authors and do not necessarily represent those of their affiliated organizations, or those of the publisher, the editors and the reviewers. Any product that may be evaluated in this article, or claim that may be made by its manufacturer, is not guaranteed or endorsed by the publisher.
